# Characteristics of patients with autoimmune haemolytic anaemia secondary to lymphoproliferative disorder: A single-centre retrospective analysis

**DOI:** 10.1038/s41598-019-56162-y

**Published:** 2019-12-23

**Authors:** Limin Xing, Manjun Zhao, Yi Wang, Yingying Feng, Yingying Qu, Ningning Duan, Yihao Wang, Huaquan Wang, Chunyan Liu, Wen Qu, Yuhong Wu, Jing Guan, Guojin Wang, Jia Song, Lijuan Li, Xiaoming Wang, Rong Fu, Zonghong Shao

**Affiliations:** 1 Haematology Department of General Hospital, Tianjin Medical University, Tianjin, 300052 China; 2 Doppler Ultrasonic Department of Tianjin Third Centre Hospital, Tianjin, 300170 China

**Keywords:** Lymphoproliferative disorders, Anaemia, Autoimmune diseases

## Abstract

Autoimmune haemolytic anaemia (AIHA) is a kind of autoimmune diseases characterized by autoantibodies which produced and secreted by abnormal activated B lymphocytes directed against red blood cells (RBC). Study reveals that about 50% AIHA mainly occurs secondary to lymphoproliferative disorders (LPD) and autoimmune diseases. In this study, we aim to explore the characteristics of patients with AIHA secondary to LPD. Fifteen patients with AIHA secondary to LPD (secondary group) and 60 with primary AIHA (primary group) were retrospectively included. Patients in the secondary group [(59.40 ± 4.74) y] were older than those in the primary group [(47.53 ± 2.30) y] (*p* = 0.024). Reticulocyte counts were lower for the secondary group [(134.55 ± 20.67) × 10^9^/L] than for the primary group [(193.88 ± 27.32) × 10^9^/L] (*p* = 0.09). Haptoglobin was higher in the secondary (0.75 ± 0.19) g/L than in the primary group (0.34 ± 0.05) g/L (*p* = 0.004). The ratio of CD3^+^CD4^+^/CD3^+^CD8^+^ was higher in the secondary (1.81 ± 0.41) than in the primary (1.05 ± 0.12) group (*p* = 0.025). Duration of remission was shorter in the secondary [(23.52 ± 5.20) months] than in the primary [(40.87 ± 3.92) months] group (*p* = 0.013). Relapse rate was higher for the secondary (33.3%) than for the primary (8.3%) group (*p* = 0.003). Mortality rate was higher in the secondary (33.3%) than in the primary (8.3%) group (*p* = 0.003). Progression-free survival was shorter in the secondary than in the primary group (*p* = 0.021). In conclusion, patients with AIHA secondary to LPD showed higher age at diagnosis, shorter remission time, and higher recurrence and mortality rates than did those with primary AIHA.

## Introduction

Autoimmune haemolytic anaemia (AIHA) is a group of heterogeneous autoimmune diseases (AD) caused by the destruction of RBC because of the presence of autoantibodies specific to RBC autoantigens. According to the aetiology, AIHA can be divided into primary and secondary. The secondary AIHA accounts for about 50% of all AIHA patients and mainly occur secondary to lymphoproliferative disorders (LPD) and autoimmune diseases^1^. The most common signs of LPD in secondary AIHA are chronic lymphocytic leukaemia (CLL) and lymphoma^2^, which can be both non-Hodgkin’s lymphoma (NHL) and Hodgkin’s lymphoma (HD). B/T cell NHL can be accompanied by AIHA, but the most common is B cell NHL (B-NHL). We compared the clinical features and response to treatment of patients with AIHA secondary to LPD with primary AIHA patients, and have summarized the characteristics of AIHA secondary to LPD in this report.

## Patients and Methods

### Patients and healthy individuals

AIHA patients were hospitalized in the Department of Haematology, Tianjin Medical University General Hospital, Tianjin, China from December 2012 to June 2016. All patients met the Chinese experts’ criteria for the diagnosis and treatment of AIHA^3^.

### Diagnosis criteria for AIHA

The criteria for the diagnosis of AIHA were as follows: the level of haemoglobin (Hb) should meet the diagnostic criteria for anaemia (Male < 120 g/L; Female < 110 g/L); the RBC autoantibodies should be detected in the patients; the results of laboratory tests should meet at least one of the following criterion— percentage of reticulocytes (Ret%) >4% or absolute value >120 × 10^9^/L; haptoglobin (Hp) < g/L; total bilirubin (TBIL) ≥ 17.1 µmol/L [indirect bilirubin (IBIL) mainly elevated]. If the patients showed a good response to glucocorticoid or even if Coombs test was negative, AIHA could also be diagnosed.

We had 15 cases in the secondary AIHA group, including eight females and seven males, with a median age of 59 years, whereas the age ranged between 14 and 87 years. Three CLL patients were in Binet Stage C, nine lymphoma patients were in Ann Arbor Stage IV, and three lymphoma patients were in Ann Arbor Stage III. The Follicular Lymphoma International Prognosis Index 2 (FLIPI-2) scores for two follicular lymphoma (FL) patients were 3. The International Prognosis Index (IPI) scores for the remaining NHL patients were recorded as follows: two cases scored 1 point, three cases scored 2 points, two cases scored 3 points, and two cases scored 4 points (Table 1). Also, 60 primary AIHA patients (27 male and 33 female) with a median age of 52 years (the age range was 14~80 years) were considered as control. The study was approved by the Ethics Committee of Tianjin Medical University General Hospital and was performed in accordance with the Declaration of Helsinki. Written informed consents were obtained from all adult patients and from the parents of child patients.

**Table 1:** International Prognosis Index (IPI) and Follicular Lymphoma IPI-2 (FLIPI-2) scoring system.

IPI		FLIPI-2	
Factor	Score	Factor	Score
Age >60 years	1	Age >60 years	1
Ann Arbor stage III-IV	1	Bone marrow invasion	1
ECOG Performance Status ≥ 2	1	Hb < 120 g/L	1
Extra nodal involved parts >1	1	Maximum diameter of LN > 6 cm	1
LDH > upper normal limit	1	Β_2_-MG > upper normal limit	1

IPI: Low risk group: 0~1; low-intermediate risk group: 2; high-intermediate risk group: 3; high risk group: 4~5.FLIPI-2: Low risk group: 0~1; intermediate risk group: 2; high risk group: ≥3.

### Clinically relevant indicators

Blood routine, reticulocyte (Ret) percentage, TBIL, IBIL, lactate dehydrogenase (LDH), complement C3, complement C4, C-reactive protein (CRP), immunoglobulin G (IgG), immunoglobulin A (IgA), immunoglobulin M (IgM), immunoglobulin E (IgE), free haemoglobin (FHb), haptoglobin (Hp), peripheral blood CD19^+^ B lymphocytes ratio, CD5^+^ B lymphocytes ratio, ratio of CD5^+^CD19^+^ to CD19^+^ and the ratio of CD4^+^ to CD8^+^ T lymphocytes were determined for all the patients.

### Treatment protocol

Basic treatment: the dose of glucocorticoids was adjusted to 0.5~1.0 mg/kg/d according to the degree of haemolysis^4^. All the patients in the secondary AIHA group received additional prednisone as part of the chemotherapy regimen. The CLL patients received CHOP chemotherapy, which included cyclophosphamide, vincristine, and prednisone. For patients with B-cell lymphoma, the chemotherapy regimens included rituximab, cyclophosphamide, doxorubicin, vincristine, and prednisone. For the treatment of T-cell lymphoma, patients received chemotherapy drugs, including fludarabine, cyclophosphamide, doxorubicin, vincristine, and prednisone. The length of the chemotherapy cycle depended on the patient’s condition.

### Definition of the therapeutic response

Complete remission (CR): Clinical symptoms disappeared. The count of RBCs, haemoglobin, and reticulocytes, percentage of reticulocytes, TBIL, and IBIL values were within the normal range; Partial remission (PR): Clinical symptoms disappeared. Hb > 80 g/L, Ret% < 4%, TBIL ≤ 34 μmol/L; No remission (NR): The condition of anaemia and haemolysis still existed after the treatments, and the laboratory test results did not meet the PR criteria. The total effective rate (OR) was the sum of CR and PR.

### Statistical analysis

All the analyses were performed with SPSS 22.0 software (SPSS, Inc., Chicago, IL USA). The results are expressed as means ± standard deviation. All the quantitative data were analysed with Student’s *t*-test and nonparametric test. Categorical data were analysed with the chi-square test. Relapse rates were estimated using the Kaplan–Meier method and the Log-rank test. Statistical differences were significant at *p* < 0.05. All the figures and graphs were made using GraphPad Prism 5 software.

## Results

### Patient characteristics

Seventy-five patients were analysed retrospectively; these included 15 patients with AIHA secondary to LPD and 60 patients with primary AIHA. Among the 15 secondary AIHA patients, three patients had CLL, 11 had NHL, which included seven B-NHL patients, four T-NHL patients, and one patient with HL. There were two diffuse large B cell lymphoma (DLBCL) patients, two FL patients, one small lymphocytic lymphoma (SLL) patient, one splenic marginal zone lymphoma (SMZL) patient, and one unclassified patient was included in B-NHL group. There were four patients with T-NHL: two with angioimmunoblastic T-cell lymphoma (AITL), one with peripheral T-cell lymphoma (PTCL), and one with anaplastic large T-cell lymphoma (ALTCL) (Table 2).

**Table 2:** The underlying disease of Secondary AIHA.

Cause for secondary AIHA	N of patients (%)
CLL (Chronic lymphocytic leukemia)	3 (20)
NHL (non-Hodgkin lymphoma)	11 (73.33)
B-cell	7 (46.67)
DLBCL (Diffuse large B-cell lymphoma)	2 (13.33)
FL (Follicular lymphoma)	2 (13.33)
SLL (small Lymphocytic lymphoma)	1 (6.67)
SMZL (splenic marginal zone lymphoma)	1 (6.67)
unclassified	1 (6.67)
T-cell	4 (26.67)
AITL (Angioimmunoblastic T-cell lymphoma)	1 (6.67)
PTCL (Peripheral T-cell lymphoma)	2 (13.33)
ALTCL (Anaplastic large T-cell lymphoma)	1 (6.67)
HD (Hodgkin disease)	1 (6.67)

### General characteristics

**Figure 1:**
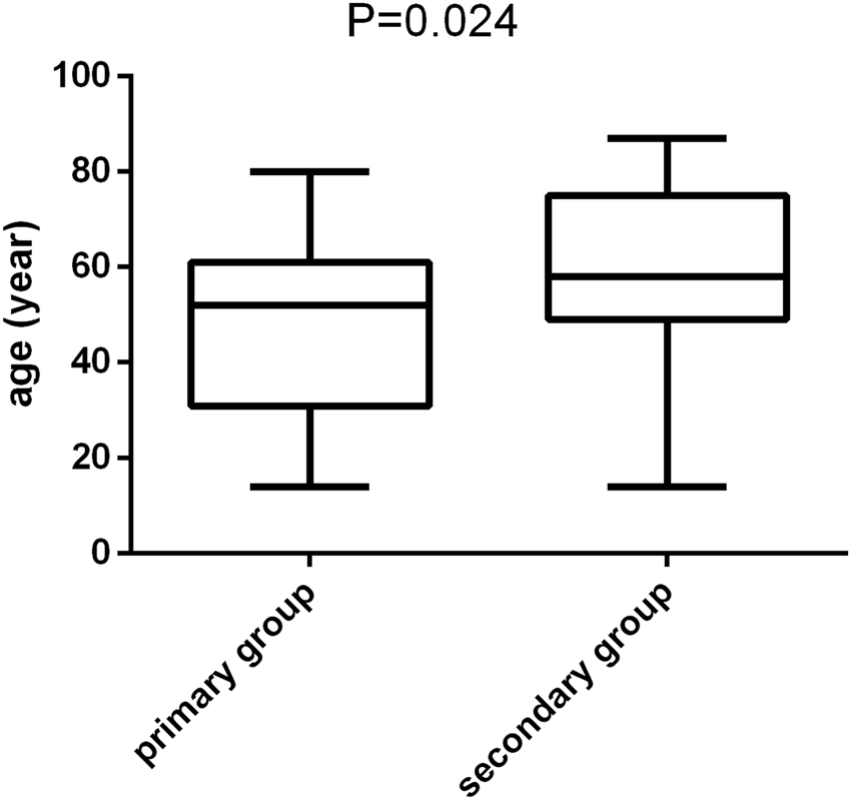
The age of secondary AIHA patients (59.40 ± 4.74 years) was older than that of primary AIHA patients (47.53 ± 2.30 years) (*p* = 0.024).

The age of the secondary AIHA patients (59.40 ± 4.74 years) was more (47.53 ± 2.30 years) than that of the primary AIHA patients (*p* = 0.024) (Table 3, Fig. 1). There were eight males and seven females in the secondary AIHA group; the ratio of male to female was 1.14. There were 60 patients (27 males and 33 females) with primary AIHA; the ratio of male to female was 0.82. The gender ratio in the two groups showed no significant difference (*p* = 1.00).

**Table 3:** Clinical characteristics of patients with AIHA at onset of the diseases.

Parameters	Secondary AIHA	Primary AIHA	*p*
Age (ys)	59.40 ± 4.74	47.53 ± 2.30	0.024*
Hb (g/L)	74.60 ± 7.43	80.45 ± 3.07	0.416
Ret (×10^9^/L)	134.55 ± 20.67	193.88 ± 27.32	0.090
TBIL (umol/l)	42.33 ± 6.45	48.35 ± 5.92	0.626
IBIL (umol/l)	30.68 ± 6.01	21.01 ± 3.21	0.177
LDH (U/L)	705.40 ± 255.23	564.15 ± 64.69	0.436
FHb (mg/L)	56.57 ± 18.76	111.88 ± 26.81	0.098
Hp (g/L)	0.75 ± 0.19	0.34 ± 0.05	0.004*

*p < 0.05.

### Haemolytic indicators

The Hb levels of patients in the secondary AIHA group [(74.60 ± 7.43) g/L] were lower than in the primary group [(80.45 ± 3.07) g/L], but there was no significant difference in the values between the two groups (*p* = 0.416). The absolute value of Ret in the secondary group [(134.55 ± 20.67) × 10^9^/L] was significantly lower than in the primary group [(193.88 ± 27.32) × 10^9^/L] (*p* = 0.09); the level of Hp in the secondary AIHA group [(0.75 ± 0.19) g/L] was higher than that in the primary AIHA group [(0.34 ± 0.05) g/L] (*p* = 0.004). The levels of FHb, LDH, TBIL, and IBIL were not significantly different between the two comparison groups (Table 3).

### Immunological features

The level of IgG in patients in the secondary AIHA group [(1113.79 ± 89.05) mg/dL] was higher than in the primary group [(1072.44 ± 75.28) mg/dL], but there was no statistical difference (*p* = 0.734). The IgA levels in the secondary and primary AIHA groups were recorded as[(178.09 ± 25.76 and 172.63 ± 16.84) mg/dL], respectively, and there was no significant difference (*p* = 0.861). The ratio of CD3^+^CD4^+^/CD3^+^CD8^+^ in the secondary AIHA group (1.81 ± 0.41) was statistically higher than in the primary group (1.05 ± 0.12) (*p* = 0.025); there were no significant differences in the IgE levels and CD5^+^CD19^+^/CD19^+^ ratio between the two groups (*p* = 0.895, *p* = 0.088, respectively) (Table 4).

**Table 4:** The Immune Characteristics at onset of the diseases.

	Secondary	Primary	*p*
C3 (mg/dl)	72.50 ± 6.47	66.90 ± 3.57	0.467
C4 (mg/dl)	16.89 ± 4.51	15.16 ± 0.94	0.558
IgG (mg/dl)	1113.79 ± 89.05	1072.44 ± 75.28	0.734
IgA (mg/dl)	178.09 ± 25.76	172.63 ± 16.84	0.861
IgM (mg/dl)	172.74 ± 54.14	135.5 ± 30.91	0.545
IgE (IU/ml)	91.9 ± 47.36	84.39 ± 30.06	0.895
CRP (mg/dl)	1.51 ± 0.56	0.97 ± 0.33	0.421
CD5^+^CD19^+^/CD19^+^ (%)	24.91 ± 10.09	5.41 ± 1.91	0.088
CD3^+^CD4^+^/CD3^+^CD8^+^ (%)	1.81 ± 0.41	1.05 ± 0.12	0.025*

*p < 0.05.

### Direct antiglobulin test (DAT)

Among all secondary AIHA patients, DAT was negative for seven patients, and one case was DAT positive for IgM and one for IgG with the complement 3. The remaining six patients did not undergo the DAT test. The patient who was DAT positive for IgM suffered from AITL (IVA stage) and died after 2 years. The patient with IgG and complement 3 had FL (IVB stage).

## Efficacy Evaluation and follow-up of Patients with AIHA Secondary to LPD and Patients with Primary AIHA

### Efficacy response

**Figure 2:**
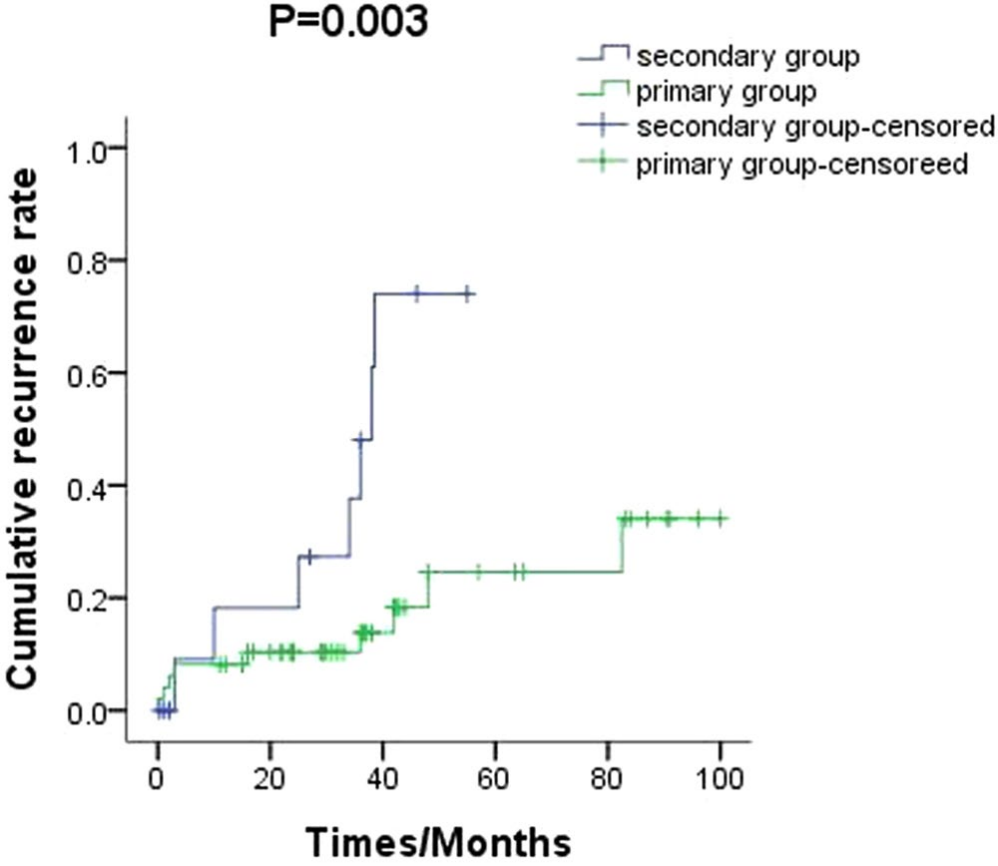
The cumulative recurrence rate of secondary AIHA patients was 33.3%, which is markedly higher than that of primary AIHA patients (8.3%) (*p* = 0.003).

The patients were treated with a corticosteroid-based regimen in both the groups: there was no statistical difference for the treatment onset time between the secondary AIHA group (25.50 ± 5.94 days) and the primary AIHA group (14.26 ± 1.134 days) (*p* = 0.094). The duration of remission in the secondary group (23.52 ± 5.20 months) was shorter than that in the primary group (40.87 ± 3.92 months) (*p* = 0.013)(Fig. 2).

### Prognosis

The median follow-up time was 36 (2~108) months. The cumulative recurrence rate in the secondary AIHA group (33.3%) was higher than in the primary group (8.3%) (*p* = 0.003) (Fig. 2). The mortality rate in the secondary AIHA group (33.3%) was higher than in the primary group (8.3%) (*p* = 0.003).

**Figure 3:**
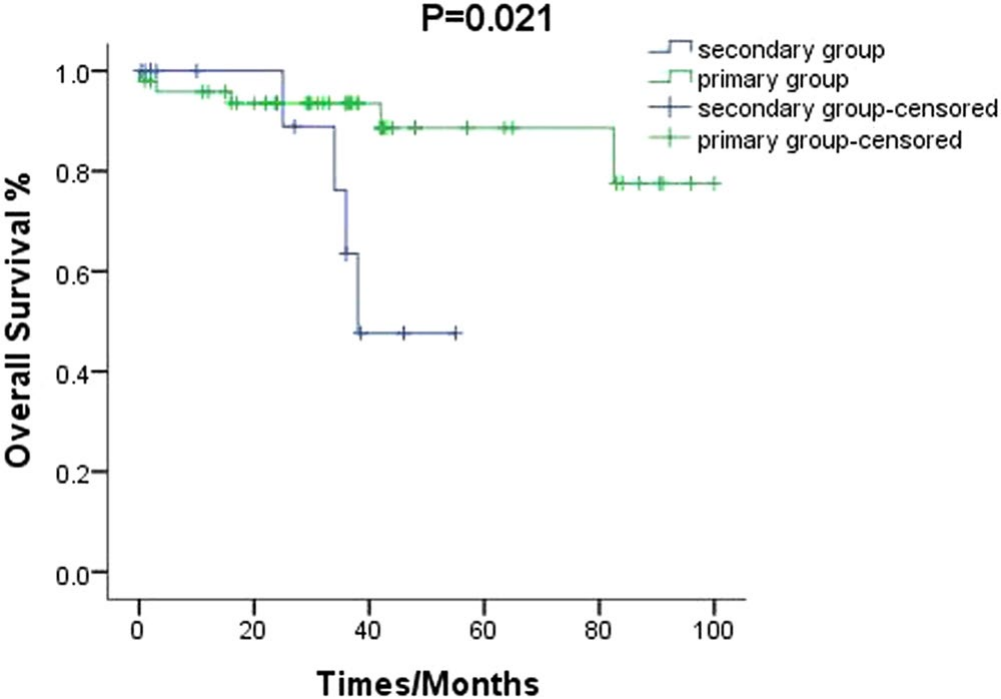
The progression-free survival of patients with secondary AIHA was significantly shorter than that of primary AIHA patients. (*p* = 0.021).

Among the 15 cases of secondary AIHA, five patients died. Among those five cases, there were three patients who instantly died from uncontrollable infection, and one patient died from intracerebral haemorrhage. One patient died of intractable haemolysis, which resulted in significant organ failure (Table 5). The progression-free survival of patients with AIHA secondary to LPD was significantly shorter than that of primary AIHA patients (*p* = 0.021) (Fig. 3).

**Table 5:** Death cases in secondary AIHA Patients.

Case	Age	Sex	Diagnose	Coombs	Cause of death	Time to relapse (months)	Total course (months)
1	78	Female	CLL	—	Infection	NR	38
2	47	Male	AITL (IV A)	IgM	Pulmonary infection	NR	25
3	74	Male	CLL	—	Encephalorrhagia	NR	36
4	75	Male	CLL	—	Infection	24	34
5	87	Female	PTCL (IV A)	—	Hemolysis	NR	2

## Discussion

AIHA is caused by the antibodies against RBCs as a result of immune dysfunction. It can be divided into primary and secondary AIHA, according to the aetiology. LPD is the common cause of secondary AIHA, among which NHL and CLL are the common primary diseases. The incidence of AIHA in patients with NHL was 0.23~6.2%^2^. The common kinds of NHL with AIHA include DLBCL, FL, small B-cell lymphoma, marginal zone lymphoma (MZL), and PTL^5,6^. The incidence of AIHA in the CLL patients is about 30%^2^. Hu retrospectively analysed 4880 patients who had been diagnosed with NHL and received treatment at Peking Union Hospital from 1995 to 2017. They found that 15.7% of the NHL patients had AIHA and among them 58.82% of B-NHL patients had DLBCL. In T-NHL secondary AIHA patients, AITL accounted for 57.14% of the cases^7^.

In our study, we found that among the LPD patients with AIHA, NHL was most commonly seen with an incidence of 73.33% (11/15) and B-NHL patients accounted for 46.67% of the cases (7/15). Moreover, FL and small B-cell NHL cases were dominant among the B-NHL patients, and accounted for nearly 50% of the cases of B-NHL. T-NHL patients accounted for 26.67% of the cases (4/15), and the lymphoma category showed no obvious tendency. Also, the percentage of T-NHL secondary AIHA patients was different from that reported by Hu. Only 20% of the CLL secondary AIHA patients were present (3/15) in our study, which may be related to the low incidence of CLL in the Asian population. The incidence rate of AIHA secondary to LPD is significantly low.

In Europe, a study with 492 patients showed that the incidence of AIHA associated with Hodgkin’s disease was 0.2%^8^ and the data from the United States was 3–4%^9^, which usually occurs in stage III and IV of nodular sclerosis and mixed cell type^10,11^. We found that only one patient had stage IV Hodgkin’s disease, and the pathological pattern showed a combined cell type.

The age of the patients in the secondary AIHA group was higher than that in the primary AIHA group (about 10 years older), and there was statistical difference between the two groups, whereas this was related to the older onset age of NHL. The ratio of male to female was similar, and there was no statistical difference between the two groups. The result was different from that reported by Zhuang Yun^12^ and Mauro^13^.

AIHA secondary to LPD is induced by different autoantibodies produced by our body. Tim^14^ proposed that the causes and mechanisms for autoimmune cytopenia (AIC) in CLL are autoantibodies produced by non-malignant B cells, and antigen presentation and secretion of cytokines by CLL cells. Changes in the microenvironment that affect the T cell pools and their characteristics also play an important role in the pathogenesis of AIHA secondary to CLL. High-risk factors for AIHA in CLL patients include progressive disease, advanced age, small-ploidy lymphocytes, high level of beta-2 microglobulin, cytogenetic abnormalities, and non-mutations of the IGHV gene^15^. Furthermore, the combination of LPD and AIHA occurred not only in B-NHL but also in T-NHL, and it is not only an inert disease but also causes invasive infection. The production of autoantibodies in NHL may be related to the failure of the body’s immune tolerance mechanism to eliminate immature autoreactive- lymphocytes, causing certain antigenic substances to be exposed on the surface of red blood cells and platelets. Autoreactive clones because of genetic abnormality (*BCL-2, C-MYC*) are activated by viral infections and other unknown events. Defects in the apoptotic pathway including FAS/FASL cause the excessive accumulation of autoreactive T cell clones^16^. Finally, immune abnormalities are the ultimate cause of AIHA. We found that the ratio of CD3^+^CD4^+^/CD3^+^CD8^+^ cells in the secondary AIHA group was higher than that in the primary AIHA group, suggesting that cellular immune abnormalities are involved in the pathogenesis of AIHA secondary to LPD. Kalpadakis found that when AIHA occurs secondary to LPD, it can still relapse when LPD achieves a complete remission, suggesting that AIHA secondary to LPD is not entirely caused by tumour cells^17^. However, in our study, relapse of LPD was seen in all the patients with recurrence of secondary AIHA. There was no significant difference in the degree of haemolysis between the two comparison groups. Although Ret in the secondary group was lower than that in the primary group and Hp in the secondary group was higher than in the primary group, the levels of FHb, IBIL, TBIL, and LDH showed no statistically significant differences, which indicates that the degree of haemolysis in the secondary group was similar to that in the primary group.

Ret is not only related to the degree of haemolysis, but also relates to the proliferation of bone marrow. In NHL patients, during the chemotherapy period, Ret may be reduced because of the influence of bone marrow hypoplasia. Hp is an acute phase protein the levels of which are affected by LPD itself.

In this group of patients, the positive rate of DAT was lower. Chadebech found that clinical severity might be dependent on the functional activity of the anti-RBC antibodies and suggested to investigate the activation state of the patients’ immune cells^18^. It is necessary to improve the sensitivity of DAT.

In addition to the first-line treatment with glucocorticoid drugs, the second-line treatment includes rituximab, immunosuppressive drugs (such as mycophenolate mofetil, MMF), splenectomy, alemtuzumab, and haematopoietic stem cell transplantation^19,20^. Patients with AIHA secondary to LPD received CHOP or CHOP-based chemotherapy according to their clinical manifestations. The treatment onset time for secondary AIHA patients was longer than that of primary AIHA, and the duration of remission of secondary group was statistically shorter than that of the primary group. This suggests that the effect of treatment on patients in the secondary group was unsatisfactory, and the prognosis was poor. The presence of AIHA is one of the indicators of poor prognosis in LPD, affecting the overall survival of LPD patients. In an analysis conducted by Giuseppe, 830 newly diagnosed CLL patients were enrolled^21^. The prognosis of patients with CLL complicated with secondary Evans syndrome was poor^21^. Among the 15 secondary AIHA patients in our study, three died from infection and one died from haemolysis. Five of the 60 patients in the primary group died; of these, two patients died from haemolysis. The relapse rate of secondary AIHA patients is high and the duration time of remission is short. We have been following 75 AIHA patients in the primary and secondary group for 108 months, and the recurrence rate of the secondary group was significantly higher than in the primary group.

In conclusion, the age of patients with AIHA secondary to LPD tends to be higher; thus, the immune disbalance tends to be increased relative to that in primary AIHA patients. Therefore, it is necessary to monitor the health condition of AIHA patients for timely diagnosis of LPD.
